# Laparoscopy experience in East, Central, and Southern Africa: insights from operative case volume analysis

**DOI:** 10.1007/s00464-024-10960-2

**Published:** 2024-06-18

**Authors:** Yves Yankunze, Michael M. Mwachiro, June Owino Lando, Niraj Bachheta, Deirdre Mangaoang, Abebe Bekele, Robert K. Parker

**Affiliations:** 1 Department of Surgery, Tenwek Hospital, PO Box 39, Bomet, 20400 Kenya; 2 College of Surgeons of East, Central, and Southern Africa, Arusha, Tanzania; 3 Institute of Global Surgery, Royal College of Surgeons in Ireland, Dublin, Ireland; 4 Department of Surgery, Alpert Medical School of Brown University, Providence, RI USA; 5 University of Global Health Equity, Kigali, Rwanda

**Keywords:** Global surgery, Laparoscopy, Minimally invasive, Surgical education, Surgical workforce, Operative case volumes

## Abstract

**Background:**

With the primary objective of addressing the disparity in global surgical care access, the College of Surgeons of East, Central, and Southern Africa (COSECSA) trains surgeons. While sufficient operative experience is crucial for surgical training, the extent of utilization of minimally invasive techniques during COSECSA training remains understudied.

**Methods:**

We conducted an extensive review of COSECSA general surgery trainees' operative case logs from January 1, 2015, to December 31, 2020, focusing on the utilization of minimally invasive surgical procedures. Our primary objective was to determine the prevalence of laparoscopic procedures and compare this to open procedures. We analyzed the distribution of laparoscopic cases across common indications such as cholecystectomy, appendicitis, and hernia operations. Additionally, we examined the impact of trainee autonomy, country development index, and hospital type on laparoscopy utilization.

**Results:**

Among 68,659 total cases, only 616 (0.9%) were laparoscopic procedures. Notably, 34 cases were conducted during trainee external rotations in countries like the United Kingdom, Germany, and India. Gallbladder and appendix pathologies were most frequent among the 582 recorded laparoscopic cases performed in Africa. Laparoscopic cholecystectomy accounted for 29% (276 of 975 cases), laparoscopic appendectomy for 3% (76 of 2548 cases), and laparoscopic hernia repairs for 0.5% (26 of 5620 cases). Trainees self-reported lower autonomy for laparoscopic (22.5%) than open cases (61.5%). Laparoscopy usage was more prevalent in upper-middle-income (2.7%) and lower-middle-income countries (0.8%) compared with lower-income countries (0.5%) (*p* < 0.001). Private (1.6%) and faith-based hospitals (1.5%) showed greater laparoscopy utilization than public hospitals (0.5%) (*p* < 0.001).

**Conclusions:**

The study highlights the relatively low utilization of minimally invasive techniques in surgical training within the ECSA region. Laparoscopic cases remain a minority, with variations observed based on specific diagnoses. The findings suggest a need to enhance exposure to minimally invasive procedures to ensure well-rounded training and proficiency in these techniques.

The global disparity in surgical access has been a pressing concern [1], with a pronounced gap evident in East, Central, and Southern Africa. To address this issue, the College of Surgeons of East, Central, and Southern Africa (COSECSA) was established to facilitate surgical training within the region [2]. While operative case experience is crucial for surgical training and has been shown to improve technical skills and work-based assessments [3, 4], the focus has predominantly been on open procedures.

Since its advancement in the 1980s, minimally invasive surgery has played an important role in general surgery. It offers benefits over open surgery with reduced hospital stays and decreased surgical site infections. Surgical training programs have been changing regarding required laparoscopic skills for trainees. For example, in the United States, the Fundamentals of Laparoscopic Surgery (FLS) certification examination is a requirement for taking the American Board of Surgery (ABS) qualifying examination [5]. The current state of minimally invasive surgery (MIS) and laparoscopy in sub-Saharan Africa is a subject of increasing importance, especially given its potential benefits and the growing interest among surgeons in the region to overcome the existing barriers to its widespread implementation. In a survey of surgeons in East, Central, and Southern Africa, 93% of respondents expressed a desire to increase their laparoscopy utilization [6]. Studies in the region have demonstrated the feasibility and utility of laparoscopy in treating pathologies [7–11]. Audits at institutions in Nigeria and Senegal highlight the advantages of laparoscopic surgery, demonstrating shorter hospital stays and equivalent wound complication rates compared with open procedures [12, 13]. Despite the evident potential, sub-Saharan Africa faces significant obstacles in incorporating laparoscopy into surgical practice, including limited access to training resources, mentors, equipment, and industry presence, and consumable supplies, as well as high costs [6, 14, 15].

Recent investigations have examined various training programs in African countries, shedding light on the diverse case volumes trainees encounter [16, 17]. There have been suggested guideline minimums for surgical procedures assigned to each trainee in the region; however, accreditation criteria currently do not account for MIS training [17, 18]. Not only is there no recommended minimum number assigned to minimally invasive surgical procedures, but also there is a limited understanding of the application of these techniques in this setup. This void in research represents an unexplored area that warrants further investigation, as a detailed understanding could offer insights into the quality of surgical training and potential avenues for improvement.

The study aims to investigate laparoscopic procedures performed by COSECSA trainees, quantifying and categorizing the types of procedures while comparing them to open techniques. This research seeks to fill the identified knowledge gap, offering a benchmark for surgical training in East, Central, and Southern Africa.

## Materials and methods

### Study design and data source

This study is an in-depth sub-analysis of operative case logs maintained by general surgery trainees under the College of Surgeons of East, Central, and Southern Africa (COSECSA) between January 1, 2015, and December 31, 2020. A previous study investigated overall operative case volumes for COSECSA trainees [17]. For the current analysis, we specifically concentrated on laparoscopic procedures performed during the training period. The COSECSA eLogbook, initiated in 2015, served as our primary data repository [19]. These studies received ethical approval from COSECSA.

### Study population

Trainees enrolled in either the FCS (Fellows of COSECSA) General Surgery program or the MCS (Members of COSECSA) program were included [20]. Our analysis excluded those who had not completed the requisite training years—two for MCS and three for FCS. Operations carried out while trainees were unenrolled were not considered. However, procedures undertaken in COSECSA unaccredited hospitals were retained to reflect the breadth of trainee experience. Cases were included regardless of geography to understand the trainee experience. However, to understand the utilization of laparoscopy within Africa, cases were excluded when not performed on the continent.

### Data collection and categorization

Laparoscopic procedures were identified and categorized according to predetermined categories set forth by COSECSA and subsequently validated. These procedures were further divided based on common indications such as cholecystectomy, appendicitis, and hernia repairs. We also integrated variables such as the level of trainee autonomy, the development index of the country where the operation was performed (as defined by the World Bank [21]), and the type of hospital facility (self-defined as public, private, or faith-based). To better understand the differences from geography and development indices, we examined case logs from each country and compared the case volumes in laparoscopy. For simplicity, the presence of autonomy was defined as a dichotomous variable with the resident’s self-reporting involvement of assisting and performing with the trainer scrubbed as no autonomy and performing the operation with the trainer unscrubbed or not present as with autonomy.

To ensure the validity of our data, multiple surgeons reviewed a random sample of procedures for categorization consistency, and agreement was assessed using Cohen's kappa statistic [17].

### Outcome measures

Our primary outcomes were the prevalence of laparoscopic procedures relative to open surgeries and the percentage of cases involving laparoscopy for trainees’ experience. Secondary outcomes included the distribution of laparoscopic procedures over time and across varying indications, as well as the influence of external variables like country development index and hospital type on case utilization. An evaluation of the trainees’ self-reported autonomy was performed, and a comparison between laparoscopic and open cases was conducted.

### Statistical analysis

Data were assembled in Microsoft Excel and analyzed with Stata version 16.0. Descriptive statistics were used to summarize the data. Nonparametric tests were employed to compare the volume of laparoscopic to open procedures and to examine variations across geographical and socio-economic settings. A p-value threshold of 0.05 was considered to indicate statistical significance.

## Results

Among 68,659 total cases, only 616 (0.9%) were laparoscopic procedures. Trainees had limited exposure with a median of 10 (IQR 6–17) laparoscopic cases for those with a complete five-year experience. Trainee experience with laparoscopy is detailed in Table 1. Ninety-five trainees (49.0%) reported no experience with an operation involving laparoscopy. Trainees reported lower autonomy for laparoscopic (22.5%) than open cases (61.5%) (*p* < 0.001).

**Table 1:** The experience of surgical trainees with laparoscopy stratified by trainees with all five years of recorded cases, trainees in their first 2 years, and trainees in their final 3 years of general surgery training

	Complete 5 years	MCS Post-graduate years 1 and 2	FCS Post-graduate years 3, 4, and 5
Number of trainees	14	177	31
Number of trainees with no lap experience (%)	1 (7.1%)	94 (53.1%)	8 (25.8%)
Median Lap cases (IQR)	10 (6,17)	0 (0.3)	4 (0.5,11.5)
Median total cases (IQR)	848 (722–998)	248 (205,360)	468 (353.5,701.5)
Median percentage of lap cases (IQR)	1.2 (0.8–2.0)	0 (0,0.8)	1.0 (0.07–2.2)

**Fig. 1:**
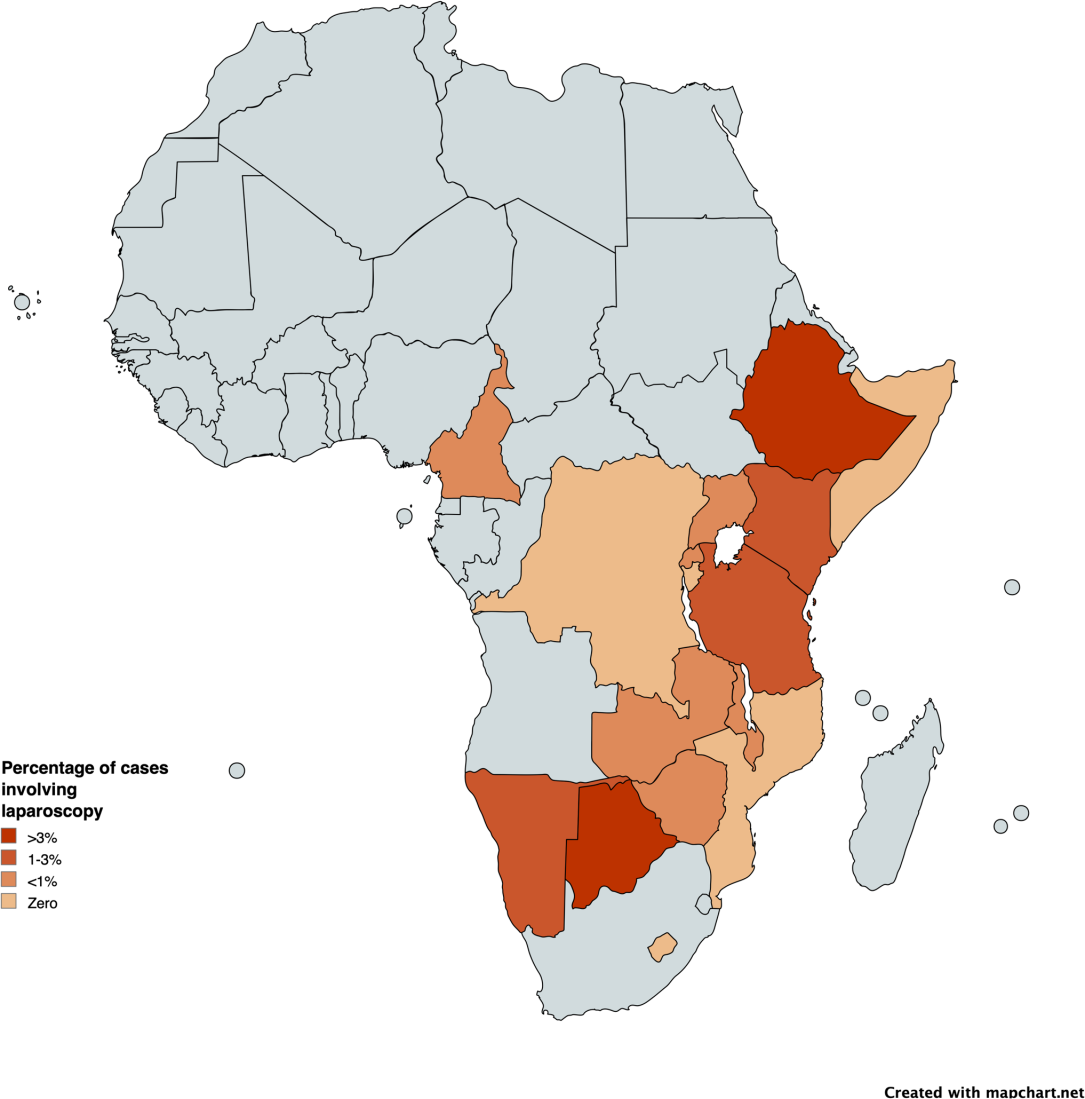
Map of countries with included COSECSA training centers with percentage of laparoscopic cases. The figure displays a map of the continent of Africa to depict the percentage of laparoscopic cases performed by COSECSA trainees. Darker shades demonstrate higher percentage of laparoscopic cases from that country. Lighter shades demonstrate lower percentage of laparoscopic cases from that country

Notably, 34 cases were conducted during trainee external rotations in countries like the United Kingdom, Germany, and India. The uptake of laparoscopy varies by country (Fig. 1) with specific numbers detailed in Table 2. Among the 582 laparoscopic cases performed in Africa, gallbladder (47%) and appendix (12%) pathologies were the most frequent. Laparoscopic cholecystectomy accounted for 29% (276 of 975 cholecystectomies), laparoscopic appendectomy for 3% (76 of 2548 appendectomies), and laparoscopic hernia repairs for 0.5% (26 of 5620 hernia repairs).

**Table 2:** Utilization of laparoscopy by Country as reported by surgical trainees

Reported Country	Number of Cases	Number of Reported Cases involving Laparoscopy
Botswana	1121	35 (3.1%)
Burundi	1292	0 (0.0%)
Cameroon	991	1 (0.1%)
The Democratic Republic of the Congo	880	0 (0.0%)
Ethiopia	1242	44 (3.5%)
Kenya	23,572	259 (1.1%)
Lesotho	340	0 (0.0%)
Malawi	8249	3 (0.0%)
Mozambique	128	0 (0.0%)
Namibia	1732	43 (2.5%)
Rwanda	4091	38 (0.9%)
Somalia	241	0 (0.0%)
Tanzania	3415	33 (1.0%)
Uganda	1607	11 (0.7%)
Zambia	11,384	38 (0.3%)
Zimbabwe	8600	77 (0.9%)

There were no apparent trends over time with the usage of laparoscopy. Laparoscopy usage was more prevalent in upper-middle-income (2.7%) and lower-middle-income countries (0.8%) compared with lower-income countries (0.5%) (*p* < 0.001). Private (1.6%) and faith-based hospitals (1.5%) showed greater laparoscopy utilization than public hospitals (0.5%) (*p* < 0.001) (Table 3).

**Table 3:** Impact of case location and year on the use of laparoscopy

Factor	Total number of cases	Number of laparoscopy cases (%)
Hospital Type		
Public	47,192	236 (0.5)
Private	3,447	54 (1.7)
Faith-based	15,705	236 (1.5)
Development Index of Country of Hospital		
Lower-income	17,634	96 (0.5)
Lower-middle-income	47,894	408 (0.8)
Upper-middle-income	2,775	78 (2.7)
Year		
2015	6,124	28 (0.5)
2016	13,300	100 (0.8)
2017	15,155	185 (1.2)
2018	10,232	67 (0.7)
2019	14,680	117 (0.8)
2020	9,394	85 (0.9)

## Discussion

Our study highlights the limited exposure to MIS during COSECSA training, with only 0.9% of total cases being laparoscopic. This rate of uptake among trainees reflects the broader challenge of low utilization of laparoscopic surgery in LMICs, particularly in East, Central, and Southern Africa. Our findings provide the baseline experience with MIS and underscore the need for curriculum enhancements to better prepare surgeons for the evolving landscape of surgical techniques.

Based on the data, the achievement of suggested operative minimums in the region appears challenging [18]. Rather than focusing solely on achieving numerical targets, alternative educational objectives, such as simulation-based training, should be considered, and are being incorporated into the curriculum [22]. While a positive correlation exists between higher case volumes and favorable patient outcomes [23–26], the surgical learning curve for a particular procedure requires a wide range from 25 to 750 operations [26]. Developing surgical autonomy is paramount for fostering competent and independent surgeons without compromising patient safety [27, 28]. While patient safety can be achieved with appropriate supervision and resident autonomy [27], the limited number of laparoscopic cases that trainees are exposed to during training suggests the need for faculty presence and oversight for laparoscopy. With limited MIS encounters during training, attention should be given to the accreditation and oversight of surgeons performing minimally invasive surgery in the region.

Several barriers to the adoption of laparoscopy have been previously identified, including financial constraints for procuring and maintaining equipment, as well as the scarcity of experienced trainers and structured training programs with validated curricula [14, 15, 29, 30]. To overcome the obstacles, various solutions have been proposed, including adapting technology to suit the context of low- and middle-income countries (LMICs), reducing the costs associated with laparoscopic equipment and maintenance, telementoring, and providing sustained training for surgeons and operating room personnel [6, 31–34]. Addressing these barriers is crucial for improving access to laparoscopic surgery and enhancing the quality of surgical training in the region. In other settings, adoption of laparoscopy has been driven by surgeons [35], training initiatives [31–33], and government policies [36]. Further exploration of the impact of patient attitudes [37], hospital and training initiatives, and government priorities could help to understand their role in the uptake of laparoscopy in the region.

Creative solutions have emerged to address the laparoscopic simulation challenge using low-cost yet effective training tools, such as inexpensive box trainers and techniques like cutting and peeling a tangerine for practicing dissection and creating haptic feedback [38–40]. Tele-proctoring, artificial intelligence to gauge competency, and free online resources offer sustainable solutions for regions with limited access to trainers [41–43]. These innovative approaches can significantly enhance the accessibility and affordability of laparoscopic training, thereby increasing the adoption of MIS in LMICs. Embracing simulation into the curriculum could offset the lack of real-world experience identified in this review. With such limited case volume experience, COSECSA could consider requiring more simulation encounters before accreditation for fellowship.

Our findings indicate that laparoscopic surgery is more common in private and faith-based hospitals. This could be due to dedicated funding and institutional support for equipment and maintenance to facilitate the appropriate infrastructure. Faith-based hospitals in Africa are pivotal in providing essential and subspecialty surgical services and often collaborate with academic institutions to address the shortage of surgical education and training in LMICs [16, 44–46]. Faith-based hospitals have increased efficiency and patient satisfaction rates, which may lead to the implementation of cost-effective strategies that improve patient-centered outcomes like laparoscopy [44, 46]. Additionally, long-term faculty trained in other settings with high rates of laparoscopy or short-term visitors who support clinical care could help to advance laparoscopy by providing the requisite expertise [47]. Strengthening partnerships between these hospitals and training programs could further facilitate the integration of laparoscopic techniques into surgical practice.

Geographic variations in laparoscopic training are evident, paralleling trends observed in endoscopic training [48]. A substantial number of trainees did not gain any laparoscopic experience, especially in Burundi, the Democratic Republic of the Congo, Mozambique, and Somalia. Curriculum amendments could incorporate more comprehensive laparoscopic training to address these gaps. One strategy entails external rotations at centers specialized in laparoscopic procedures, which would augment technical skills and enrich the educational experience without jeopardizing patient safety.

Our study has limitations, including the absence of case log data from Pan-African Academy of Christian Surgeons (PAACS) trainees and the potential impact of the COVID-19 pandemic on case volumes [49]. The exclusion of PAACS trainees, who are trained in faith-based hospitals and have an independent case log system, may have led to an underestimation of laparoscopic case exposure as previous reports indicate laparoscopic cases were twice the number found in this study with about two percent of total cases [16]. The global COVID-19 pandemic has disrupted surgical training and practice worldwide [49], which may have further influenced the availability and utilization of laparoscopic procedures during the study period. These limitations highlight the need for further research to assess their effects on surgical training and to explore strategies for overcoming these challenges. Additionally, our data depend solely on the case logs entered by trainees, which may not be exhaustive or entirely accurate. While the lack of external validation is a limitation, the requisite review of case logs by program directors does introduce some level of accountability. This constraint can affect the generalizability of our findings but does not detract from the insights our study provides. Future investigations should focus on evaluating various aspects of surgical training, including but not limited to the quality of laparoscopic equipment, faculty expertise, and institutional barriers. Such inquiry could inform curriculum development aimed at integrating minimally invasive procedures more effectively into general surgical training.

The limited exposure to MIS for COSECSA trainees underscores the need for enhanced understanding and collaboration among residents, surgeons, policymakers, and stakeholders. Addressing the barriers to laparoscopic training and implementing effective training programs are essential for improving surgical care in the East, Central, and Southern Africa region. By embracing innovative training methods and fostering partnerships, the surgical community can advance the adoption of minimally invasive techniques and ultimately enhance patient outcomes.
